# The Unexpected Formation of a Broncho-Esophageal Fistula of the Right Main Stem Bronchus Status Post Esophageal Stent Placement: A Case Report

**DOI:** 10.7759/cureus.21641

**Published:** 2022-01-26

**Authors:** Harsh Patel, Ranbir Singh, Navim Mobin

**Affiliations:** 1 Internal Medicine, NewYork-Presbyterian Brooklyn Methodist Hospital, Brooklyn, USA; 2 Gastroenterology, NewYork-Presbyterian Brooklyn Methodist Hospital, Brooklyn, USA

**Keywords:** self-expandable metal stent, bronchoscopy, esophageal stent, small-cell lung carcinoma, tracheo-esophageal fistula, broncho-esophageal fistula

## Abstract

This case presents a patient with a history of non-small cell lung carcinoma who had radiation therapy complicated by esophageal dysphagia. She had a fully covered self-expanding metal stent (SEMS) placed one year ago in the proximal region of her esophagus prior to this admission. She presented to the emergency department (ED) for dyspnea on exertion. Imaging showed a persistent right lower lobe opacity, and bronchoscopy revealed a right broncho-esophageal fistula (BEF). Further investigation by endoscopy found that the fully covered SEMS migrated distally and caused the formation of her fistula. This case presents a patient with a right BEF caused by a migrating esophageal stent.

## Introduction

Broncho-esophageal fistulas (BEFs) and tracheo-esophageal fistulas (TEFs) are pathological fistulas connecting the trachea or bronchi to the esophagus. Although a rare finding, over 50% of fistulas are attributed to complications of esophageal or lung malignancy and carry a high risk of morbidity and mortality. Approximately 5%-15% of individuals with esophageal malignancy develop TEF, while about 1% of those with bronchogenic carcinoma develop a fistula [1]. Patients often present with frequent coughing after solid or liquid food intake, recurrent episodes of aspiration and pneumonia, or unexplained malnutrition [1]. Other less common causes include prolonged endotracheal intubation, surgical or endoscopic interventions, or infectious diseases such as tuberculosis [1]. Diagnosis can be aided by obtaining a contrast-enhanced esophagography that demonstrates displacement of barium contrast into the lung, or by direct visualization via bronchoscopy or endoscopy. There have been few studies exploring the complication of fistulas after esophageal stent placement. In one retrospective study, 4% of the nearly 400 patients studied after stent placement developed a fistula after a median of five months [2]. In another study, 9% of the nearly 200 patients developed fistulas, the majority of whom had radiation therapy after receiving a stent [3]. We present the case of a patient developing a BEF due to her stent migrating distally.

## Case presentation

**Figure 1:**
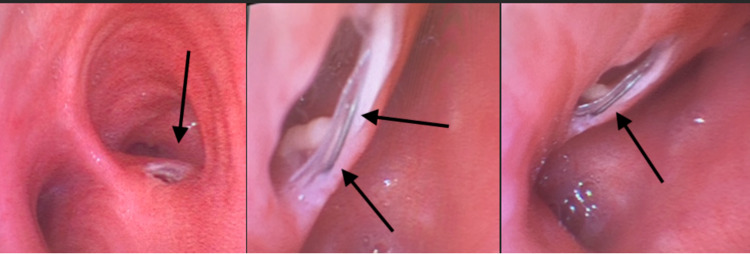
Flexible bronchoscopy image demonstrating right and left mainstem bronchus is seen in the first image. In the second image, a right mainstem bronchus with the 3 mm fistulous opening is seen with direct visualization of the esophageal stent (black arrows). The third image shows another visualization of the metallic esophageal stent during flexible bronchoscopy (black arrow).

We present the case of a 67-year-old female with past medical history significant for non-small cell lung cancer status post right lower lobe lobectomy who presented to the emergency department with two days of dyspnea on exertion. Prior to the onset of dyspnea, she had a history of nausea and vomiting for the past week and an inability to tolerate oral intake. Her oncological history is further complicated by esophageal dysphagia secondary to radiation therapy for which she had a covered self-expandable metal stent (SEMS) placed at the proximal esophagus one year prior to admission. The patient was also noted to have a recent hospitalization two months prior at an outside hospital for aspiration pneumonia requiring intubation. She was mechanically intubated for one week and successfully extubated. On initial presentation to the emergency department, the patient was noted to be hypoxic to 84% with tachypnea (respiratory rate 30) and was subsequently placed on BiPAP. One day after admission, the patient was intubated for worsening hypoxia and transferred to the intensive care unit. On day four of intubation, the patient was noted to have a persistent right lower lobe opacity despite being on broad-spectrum antibiotics, and thus a bedside bronchoscopy was performed to evaluate the lungs. A 3-mm right BEF was visualized with clear visualization of the previously placed fully covered SEMS displaced towards the mid esophagus (Figure 1).

The advanced gastrointestinal team was contacted, and the stent was replaced. A partially covered SEMS (20 mm x 10 cm) stent was placed with the proximal end 25 cm from incisors. Simultaneously, the patient’s PEG tube was replaced with a Gastrostomy-Jejunostomy tube to facilitate feeding in the setting of TEF. A repeat bronchoscopy was performed the following day which demonstrated resolution of the fistulous tract. On subsequent days, the patient was unable to be weaned off mechanical ventilation successfully. Given her poor prognosis and overall debility, the family opted to make patient comfort care and perform palliative extubation.

## Discussion

Esophageal stents are a non-surgical alternative for palliative dysphagia and are commonly indicated in those with malignancy-related extrinsic or intrinsic compression of the esophagus. Esophageal stents help to alleviate cough and dysphagia, allowing patients to maintain adequate oral intake. A TEF is a well-known severe complication that typically occurs after a median of five months once an esophageal stent is placed. Other common complications include bleeding, stent migration, severe gastroesophageal reflux disease (GERD), or stent occlusion [4]. Our patient underwent palliative esophageal stent placement, which was further complicated by multiple episodes of aspiration pneumonia, likely in the setting of undiagnosed BEF due to stent migration. 

There has been another report of BEF formed due to esophageal stent placement. In one case report by Kumar et al. [5], they had a patient who developed a BEF after having a silicone tracheobronchial Y-stent placed for tracheal stenosis. The fistula was likely formed from a misplaced stent. The fistula was repaired, and the stent was replaced with no further complications or misplacements. 

In another case report by Kimura et al., although not a BEF, their team describes a patient found to have postprandial obstruction who developed a TEF. The collapse of the patient’s damaged tracheal stent towards the esophageal side was thought to be the cause of TEF [6]. The stent was removed, and the fistula was repaired. It appears in both cases that stent migration was the primary cause of fistula formation. 

There are four main types of stents: self-expandable plastic stents (SEPS), uncovered SEMS, partially covered SEMS, and fully covered SEMS [7]. In a study by Vakil et al. involving 62 patients who were randomized to partially covered SEMS or uncovered SEMSs, it was found that stent migration rates were higher in partially covered stents vs. uncovered stents at 12% vs. 7%, respectively [8]. Given that the previous two case reports mentioned had patients developed either a BEF or TEF because of stent migration, uncovered stents could be more superior at preventing the formation of these fistulas. The uncovered SEMS allow the stent to embed in the tissue and aim to prevent migration, however, there is a significant risk of dysphagia due to tumor ingrowth [8]. SEPS have been shown to have a high migration rate in one prospective trial in 101 patients with an unresectable esophageal carcinoma by Conio et al. [9]. The study compared SEPSs to partially covered SEMSs and found the migration rates to be 29% and 17%, respectively. Overall, the investigators conclude that SEPS were the least preferable stent in this patient group [9]. The patient in this case report initially had a fully covered SEMS, which was found to have migrated towards the mid esophagus and eventually the development of a BEF. This stent was subsequently replaced by a partially covered SEMS to repair the BEF, with the goal of decreasing stent migration and preventing recurrence of a fistula.

Current management guidelines recommend combined stenting (airway and esophageal) if the lesion is in the mid to proximal esophagus. Double stenting with an airway stent is indicated due to possible airway compromise. If the fistula is located more distally, there is a decreased risk of airway stenosis and thus only an esophageal stent is indicated [1]. This case demonstrates the importance of early recognition of communicating fistulas in those with prior esophageal stents and taking this into consideration in patients presenting with aspiration pneumonia. Early recognition of this unique complication can help improve quality of life, decrease hospital length of stay, and aim to reduce mortality.

## Conclusions

Esophageal stents are the usual non-surgical route taken to manage patients with dysphagia palliatively. Although they are helpful with symptomatic management, there are many complications including TEFs or BEFs from luminal erosion and stent migration. Our case aims to highlight these complications and to urge physicians to consider this in their differential in patients with signs of aspiration pneumonia with prior stent placement. Partially covered SEMS have been studied to have lower rates of complications and thus mitigate this risk of fistula formation. When available, gastroenterologists should aim to place partially covered SEMS to reduce morbidity and mortality in individuals with radiation-induced dysphagia.
